# Supplementary material for: Luminescent Hybrid Material Based on Boron Organic Phosphor and Silica Aerogel Matrix
Source: Molecules. 2022 Aug 16;27(16):5226. doi: 10.3390/molecules27165226 (PMC9416728; doi:10.3390/molecules27165226)
Supplement: Supplementary file 1 [file molecules-27-05226-s001.zip › molecules-1847245-supplementary-updated.pdf]

molecules

MDPI

# Supplementary Materials

## Luminescent hybrid material based on boron organic phosphor and silica aerogel matrix

Roman Avetisov, Artem Lebedev, Ekaterina Suslova, Ksenia Kazmina, Kristina Runina, Vlada Kovaleva, Andrew Khomyakov, Artem Barkanov, Marina Zykova, Olga Petrova, Alisa Mukhsinova, Denis Shepel, Artyom Astafiev, Natalia Menshutina and Igor Avetissov

**Table S1.** Concentration of impurities in aerogel determined by ICP-MS analysis.

| Element | | SiO2-B-0.01 % | | SiO2-B-0.05 % | | SiO2-B-0.1 % |
|---|---|---|---|---|---|---|
| Li | < | $3.2 \times 10^{-6}$ | < | $4.5 \times 10^{-6}$ | < | $4.0 \times 10^{-6}$ |
| Be | < | $5.3 \times 10^{-6}$ | < | $7.5 \times 10^{-6}$ | < | $6.7 \times 10^{-6}$ |
| B | | **$1.4 \times 10^{-2}$** | | **$2.7 \times 10^{-2}$** | | **$4.3 \times 10^{-2}$** |
| Na | | $2.2 \times 10^{-4}$ | | $2.1 \times 10^{-4}$ | | $2.0 \times 10^{-4}$ |
| Mg | | $3.0 \times 10^{-4}$ | | $3.6 \times 10^{-4}$ | | $2.2 \times 10^{-4}$ |
| Al | < | $5.1 \times 10^{-6}$ | < | $7.2 \times 10^{-6}$ | < | $6.5 \times 10^{-6}$ |
| Si | | matrix | | matrix | | matrix |
| K | < | $3.5 \times 10^{-5}$ | < | $5.0 \times 10^{-5}$ | < | $4.5 \times 10^{-5}$ |
| Ca | | SiO+ | | SiO+ | | SiO+ |
| Sc | | $3.7 \times 10^{-4}$ | | $2.7 \times 10^{-4}$ | | $2.7 \times 10^{-4}$ |
| Ti | | $5.4 \times 10^{-4}$ | | $4.9 \times 10^{-4}$ | | $3.6 \times 10^{-4}$ |
| V | | $2.3 \times 10^{-6}$ | < | $1.1 \times 10^{-6}$ | < | $1.0 \times 10^{-6}$ |
| Cr | | $2.4 \times 10^{-4}$ | | $2.7 \times 10^{-4}$ | | $2.4 \times 10^{-4}$ |
| Mn | | $9.3 \times 10^{-5}$ | | $6.9 \times 10^{-5}$ | | $8.7 \times 10^{-6}$ |
| Fe | | $6.2 \times 10^{-4}$ | < | $2.6 \times 10^{-4}$ | < | $2.3 \times 10^{-4}$ |
| Co | | $4.5 \times 10^{-6}$ | < | $2.9 \times 10^{-6}$ | < | $2.6 \times 10^{-6}$ |
| Ni | | $9.4 \times 10^{-5}$ | | $6.4 \times 10^{-5}$ | | $3.1 \times 10^{-5}$ |
| Cu | | $1.5 \times 10^{-4}$ | | $1.2 \times 10^{-4}$ | | $3.1 \times 10^{-5}$ |
| Zn | | $5.3 \times 10^{-4}$ | | $4.0 \times 10^{-4}$ | | $3.9 \times 10^{-4}$ |
| Ga | | $1.6 \times 10^{-5}$ | | $7.3 \times 10^{-6}$ | | $5.6 \times 10^{-6}$ |
| Ge | < | $7.9 \times 10^{-7}$ | < | $1.1 \times 10^{-6}$ | < | $1.0 \times 10^{-6}$ |
| As | | $3.9 \times 10^{-6}$ | < | $1.1 \times 10^{-6}$ | < | $1.0 \times 10^{-6}$ |
| Se | < | $1.6 \times 10^{-5}$ | < | $2.2 \times 10^{-5}$ | < | $2.0 \times 10^{-5}$ |
| Rb | | $2.6 \times 10^{-5}$ | | $2.2 \times 10^{-5}$ | | $2.7 \times 10^{-5}$ |
| Sr | | $7.5 \times 10^{-6}$ | | $3.8 \times 10^{-6}$ | | $1.3 \times 10^{-6}$ |
| Y | < | $7.9 \times 10^{-8}$ | < | $1.1 \times 10^{-7}$ | < | $1.0 \times 10^{-7}$ |
| Zr | | $2.6 \times 10^{-4}$ | | $2.2 \times 10^{-4}$ | | $2.7 \times 10^{-5}$ |
| Nb | | $7.5 \times 10^{-7}$ | < | $7.5 \times 10^{-8}$ | | $2.9 \times 10^{-5}$ |
| Mo | < | $7.5 \times 10^{-5}$ | < | $1.1 \times 10^{-4}$ | < | $9.5 \times 10^{-5}$ |
| Ru | | $1.6 \times 10^{-7}$ | | $1.3 \times 10^{-6}$ | | $1.0 \times 10^{-6}$ |
| Rh | < | $5.3 \times 10^{-8}$ | < | $7.5 \times 10^{-8}$ | < | $6.7 \times 10^{-8}$ |
| Pd | < | $7.9 \times 10^{-7}$ | < | $1.1 \times 10^{-6}$ | < | $1.0 \times 10^{-6}$ |

| | | | | | | |
|---|---|---|---|---|---|---|
| Ag | | $1.7 \times 10^{-7}$ | | $5.8 \times 10^{-7}$ | | $1.4 \times 10^{-7}$ |
| Cd | | $2.0 \times 10^{-6}$ | < | $1.3 \times 10^{-6}$ | < | $1.1 \times 10^{-6}$ |
| In | < | $7.9 \times 10^{-8}$ | < | $1.1 \times 10^{-7}$ | < | $1.0 \times 10^{-7}$ |
| Sn | | $1.2 \times 10^{-5}$ | | $1.2 \times 10^{-5}$ | | $1.1 \times 10^{-5}$ |
| Sb | | $3.6 \times 10^{-6}$ | | $4.1 \times 10^{-6}$ | | $3.7 \times 10^{-6}$ |
| Te | | $2.2 \times 10^{-5}$ | | $1.8 \times 10^{-5}$ | | $1.1 \times 10^{-5}$ |
| Cs | < | $2.9 \times 10^{-7}$ | < | $4.1 \times 10^{-7}$ | < | $3.7 \times 10^{-7}$ |
| Ba | | $2.3 \times 10^{-4}$ | | $1.4 \times 10^{-4}$ | | $6.1 \times 10^{-5}$ |
| La | | $4.7 \times 10^{-8}$ | | $4.0 \times 10^{-8}$ | | $1.1 \times 10^{-7}$ |
| Ce | < | $1.2 \times 10^{-5}$ | < | $1.7 \times 10^{-5}$ | < | $1.5 \times 10^{-5}$ |
| Pr | < | $1.0 \times 10^{-6}$ | < | $1.5 \times 10^{-6}$ | < | $1.3 \times 10^{-6}$ |
| Nd | < | $4.5 \times 10^{-7}$ | < | $6.4 \times 10^{-7}$ | < | $5.7 \times 10^{-7}$ |
| Sm | < | $2.6 \times 10^{-8}$ | < | $3.8 \times 10^{-8}$ | < | $3.4 \times 10^{-8}$ |
| Eu | | $4.7 \times 10^{-8}$ | | $9.3 \times 10^{-8}$ | < | $3.4 \times 10^{-8}$ |
| Gd | < | $7.9 \times 10^{-8}$ | < | $1.1 \times 10^{-7}$ | < | $1.0 \times 10^{-7}$ |
| Tb | < | $6.9 \times 10^{-7}$ | < | $9.8 \times 10^{-7}$ | < | $8.8 \times 10^{-7}$ |
| Dy | < | $7.9 \times 10^{-7}$ | < | $1.1 \times 10^{-6}$ | < | $1.0 \times 10^{-6}$ |
| Ho | < | $7.9 \times 10^{-7}$ | < | $1.1 \times 10^{-6}$ | < | $1.0 \times 10^{-6}$ |
| Er | < | $5.3 \times 10^{-8}$ | < | $7.5 \times 10^{-8}$ | < | $6.7 \times 10^{-8}$ |
| Tm | < | $2.6 \times 10^{-8}$ | < | $3.8 \times 10^{-8}$ | < | $3.4 \times 10^{-8}$ |
| Yb | < | $2.6 \times 10^{-8}$ | < | $3.8 \times 10^{-8}$ | < | $3.4 \times 10^{-8}$ |
| Lu | < | $1.6 \times 10^{-7}$ | < | $2.3 \times 10^{-7}$ | < | $2.0 \times 10^{-7}$ |
| Hf | < | $5.3 \times 10^{-8}$ | | $1.1 \times 10^{-7}$ | < | $6.7 \times 10^{-8}$ |
| Ta | < | $2.1 \times 10^{-7}$ | < | $3.0 \times 10^{-7}$ | < | $2.7 \times 10^{-7}$ |
| W | | $8.2 \times 10^{-6}$ | | $5.9 \times 10^{-6}$ | < | $1.6 \times 10^{-6}$ |
| Re | < | $2.6 \times 10^{-8}$ | < | $3.8 \times 10^{-8}$ | < | $3.4 \times 10^{-8}$ |
| Os | < | $2.6 \times 10^{-8}$ | < | $3.8 \times 10^{-8}$ | < | $3.4 \times 10^{-8}$ |
| Ir | < | $2.6 \times 10^{-8}$ | < | $3.8 \times 10^{-8}$ | < | $3.4 \times 10^{-8}$ |
| Pt | < | $1.3 \times 10^{-7}$ | < | $1.9 \times 10^{-7}$ | < | $1.7 \times 10^{-7}$ |
| Au | < | $5.3 \times 10^{-6}$ | < | $7.5 \times 10^{-6}$ | < | $6.7 \times 10^{-6}$ |
| Hg | < | $6.5 \times 10^{-6}$ | < | $9.2 \times 10^{-6}$ | < | $8.2 \times 10^{-6}$ |
| Tl | | $2.9 \times 10^{-7}$ | | $1.5 \times 10^{-7}$ | < | $1.0 \times 10^{-7}$ |
| Pb | | $1.1 \times 10^{-5}$ | | $8.2 \times 10^{-6}$ | | $4.9 \times 10^{-6}$ |
| Bi | < | $2.1 \times 10^{-6}$ | < | $2.9 \times 10^{-6}$ | < | $2.6 \times 10^{-6}$ |
| Th | < | $1.9 \times 10^{-6}$ | < | $2.7 \times 10^{-6}$ | < | $2.5 \times 10^{-6}$ |
| U | < | $2.6 \times 10^{-8}$ | | $6.6 \times 10^{-8}$ | < | $3.4 \times 10^{-8}$ |

Sign "<" indicates the limit of determination of the element for the certain analysis.

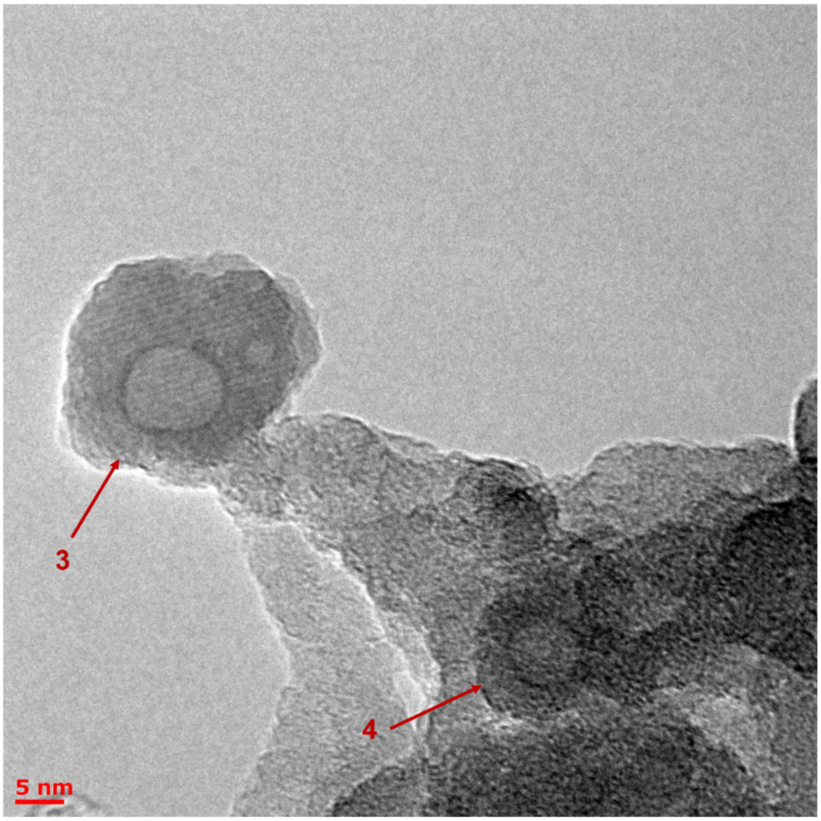


**Figure S1.** TEM image of Object 3. Shell clusters. The lattice parameter for Object 3 is 4.2 A. For Object 4, the parameter is not defined. Object diameter 3 - 12 nm (core - 6 nm), Object diameter 4 - 8 nm (core - 4 nm).

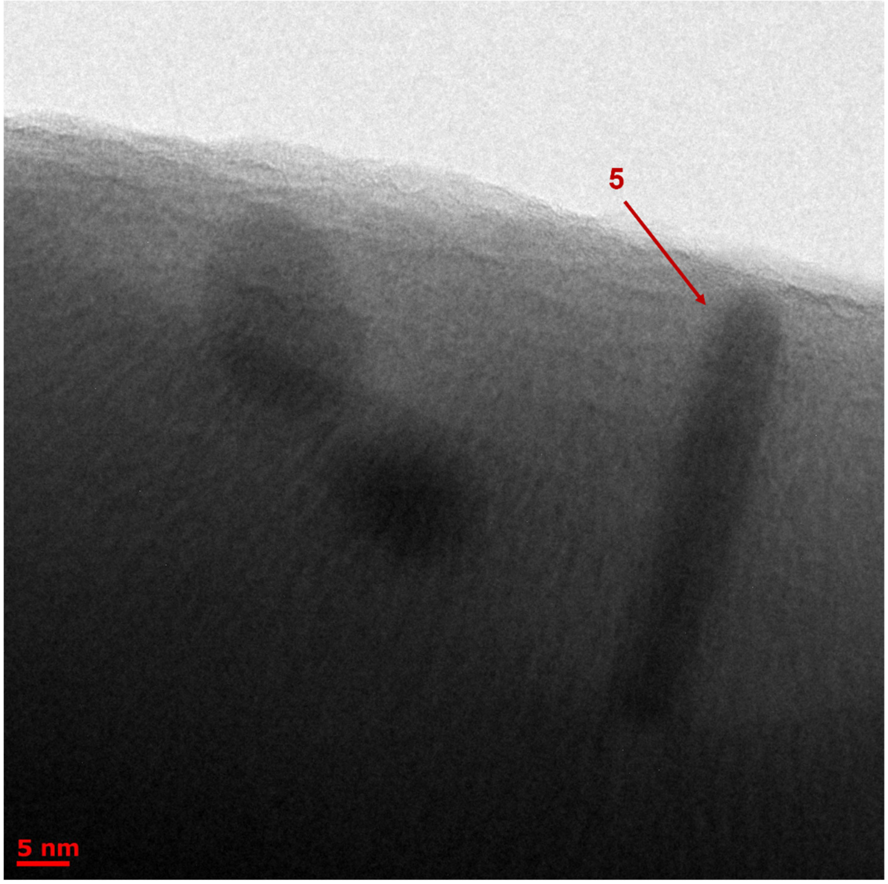


**Figure S2.** TEM image of Object 5 is a nanotube. Length - 25 nm, diameter - 5 nm

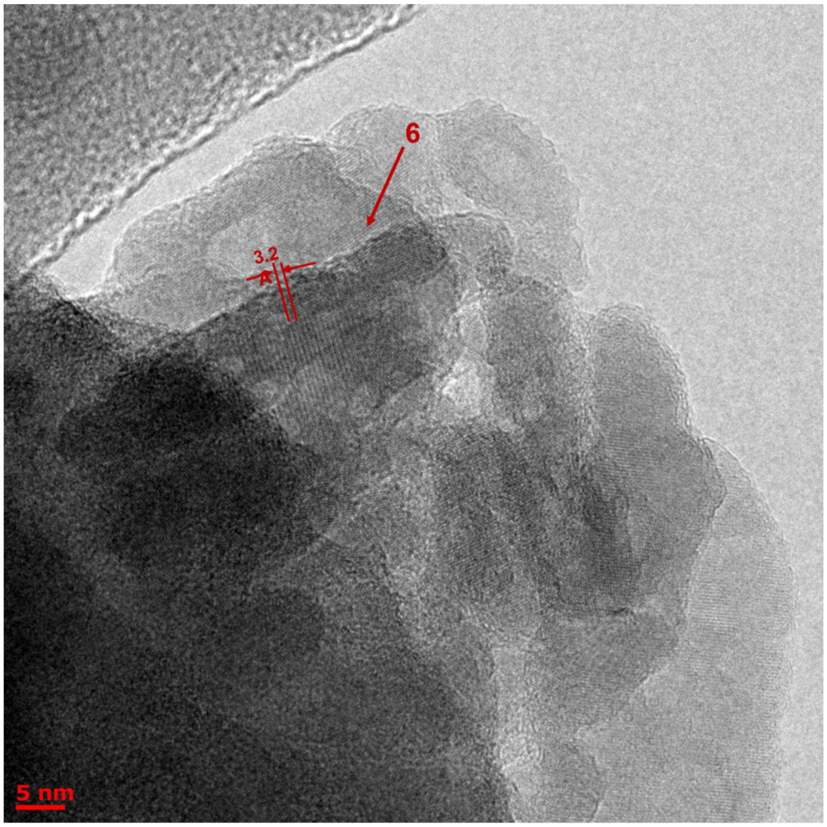


**Figure S3.** TEM image of Object 6 - several crystalline planes, partially twisted at the ends. The lattice parameter for all objects in the image is 3.2 A.

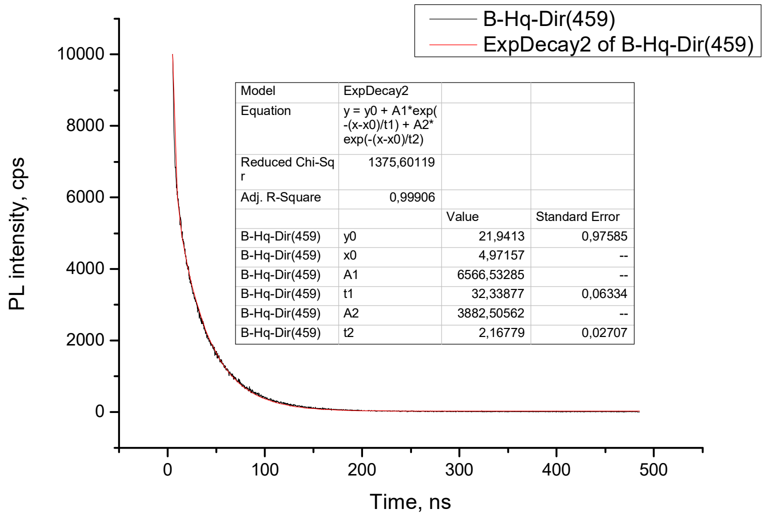


**Figure S4.** PL decay kinetics of B-Hq-Dir(459) sample obtained by the direct synthesis.

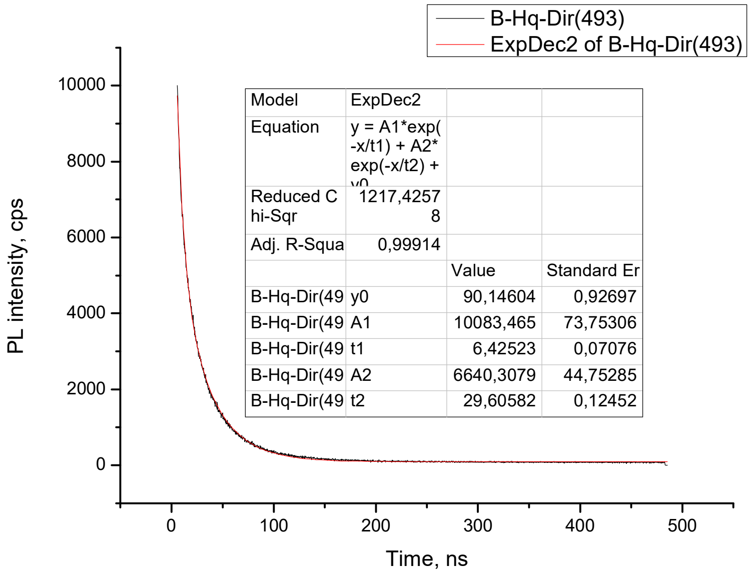


**Figure S5.** PL decay kinetics of B-Hq-Dir(493) sample obtained by the direct synthesis.

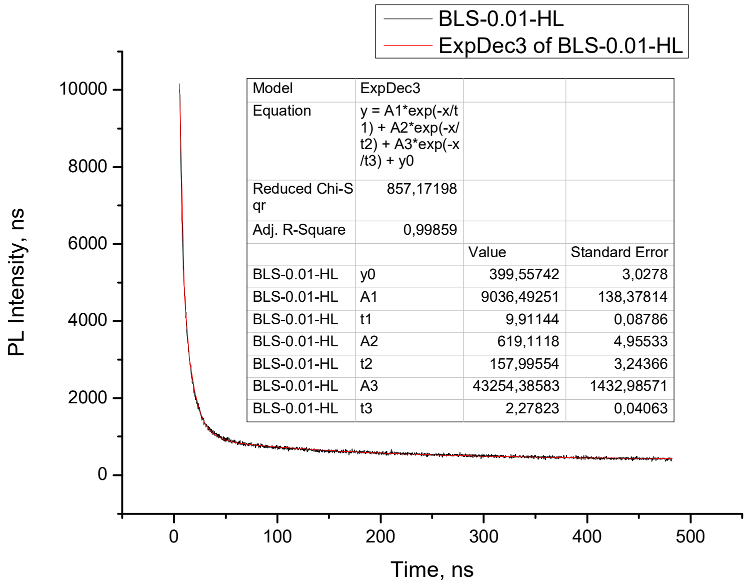


**Figure S6.** PL decay kinetics of BLS-0.01-HL sample.

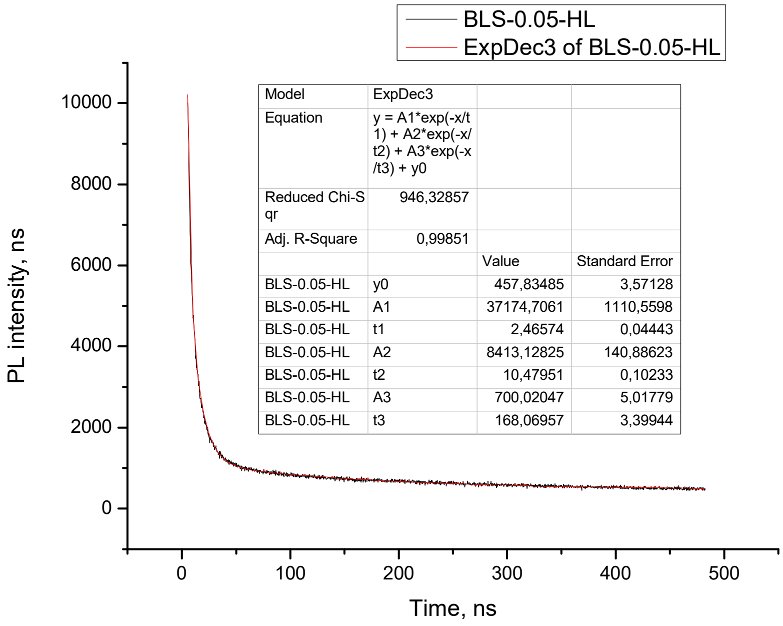


**Figure S7.** PL decay kinetics of BLS-0.05-HL sample.

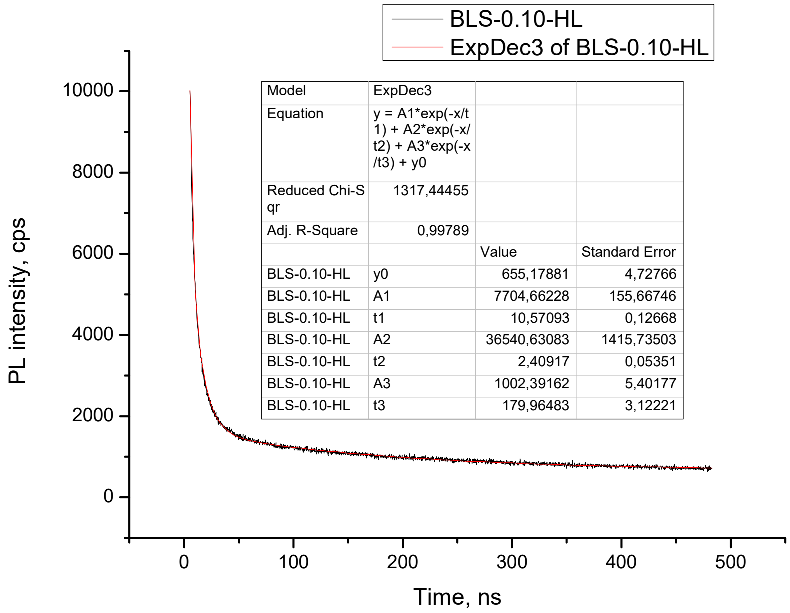


**Figure S8.** PL decay kinetics of BLS-0.10-HL sample.

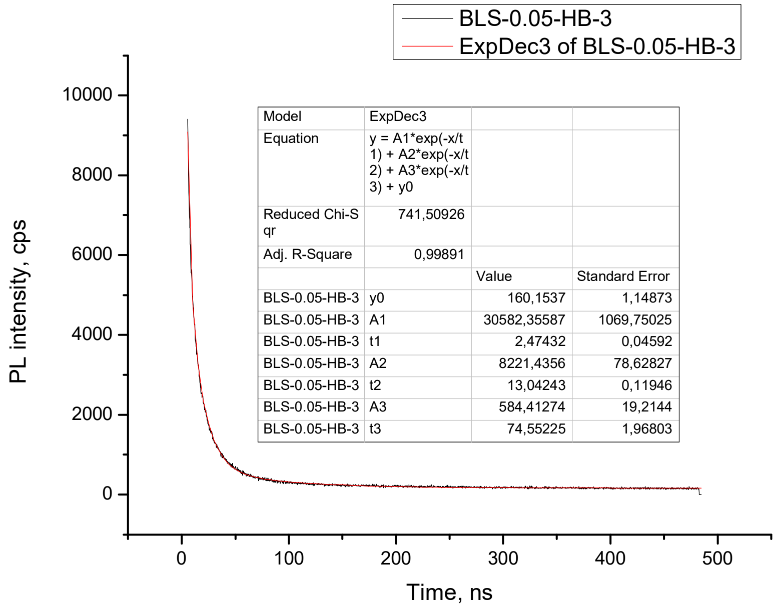


**Figure S9.** PL decay kinetics of BLS-0.05-HB-3 sample.

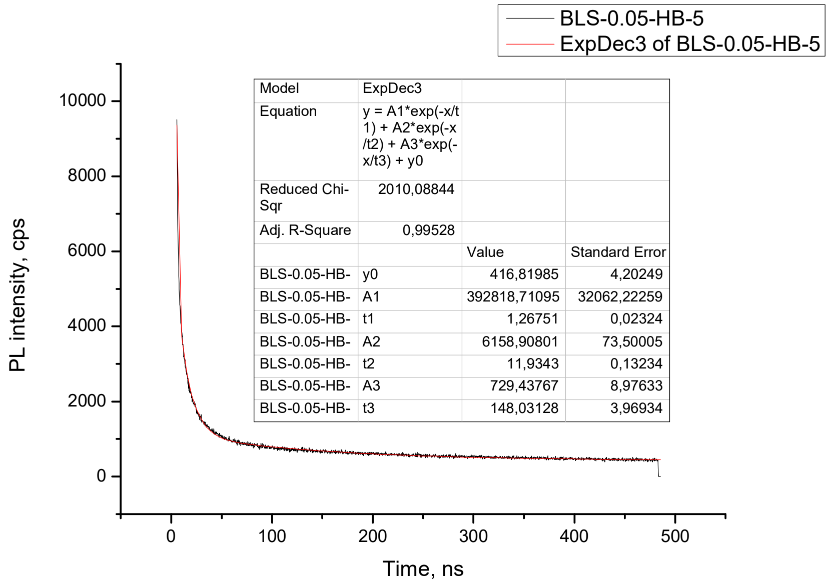


**Figure S10.** PL decay kinetics of BLS-0.05-HB-3 sample.

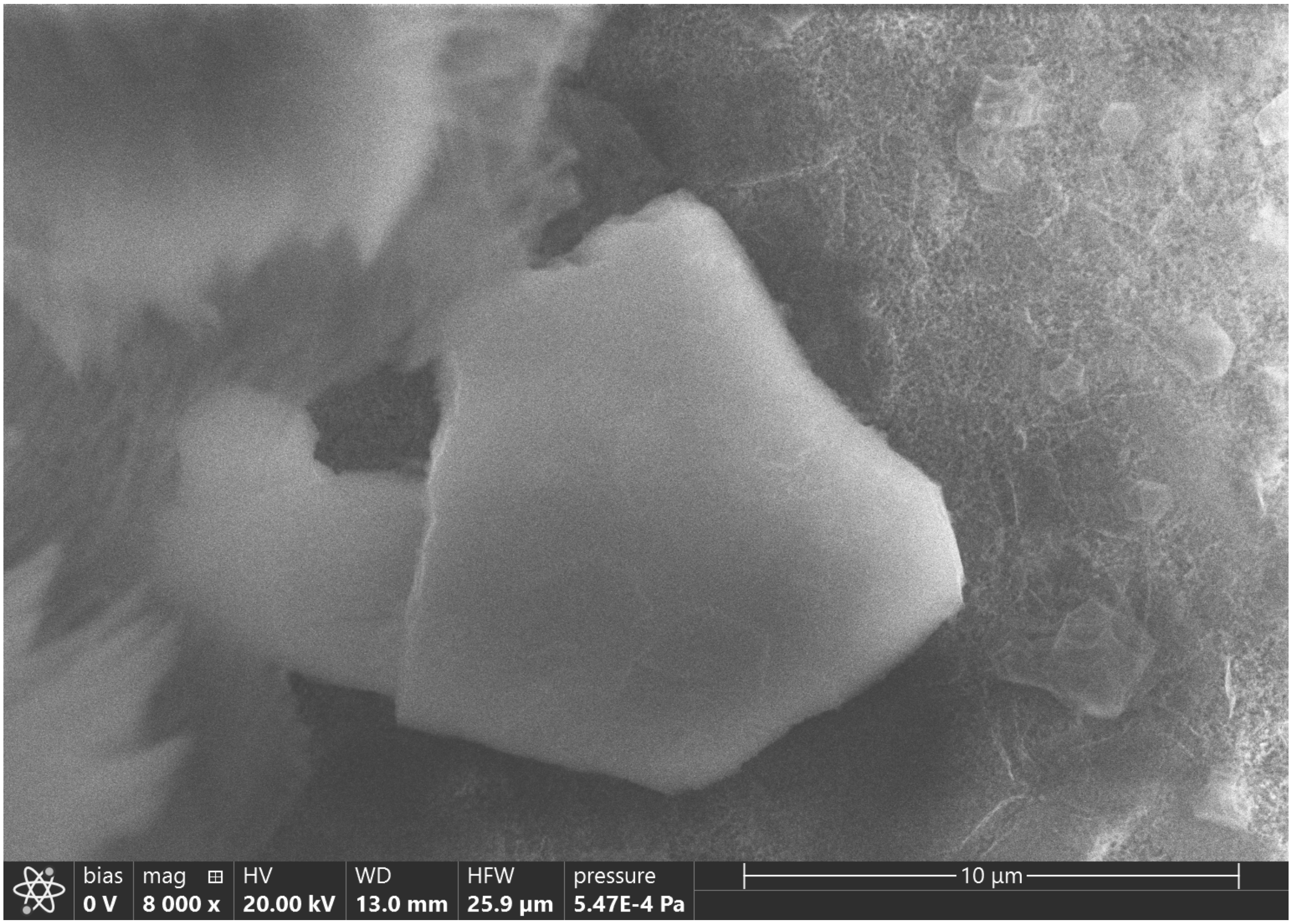


**Figure S11.** SEM image of BLC preparation fabricated by the direct synthesis at 20 kV accelerating voltage.

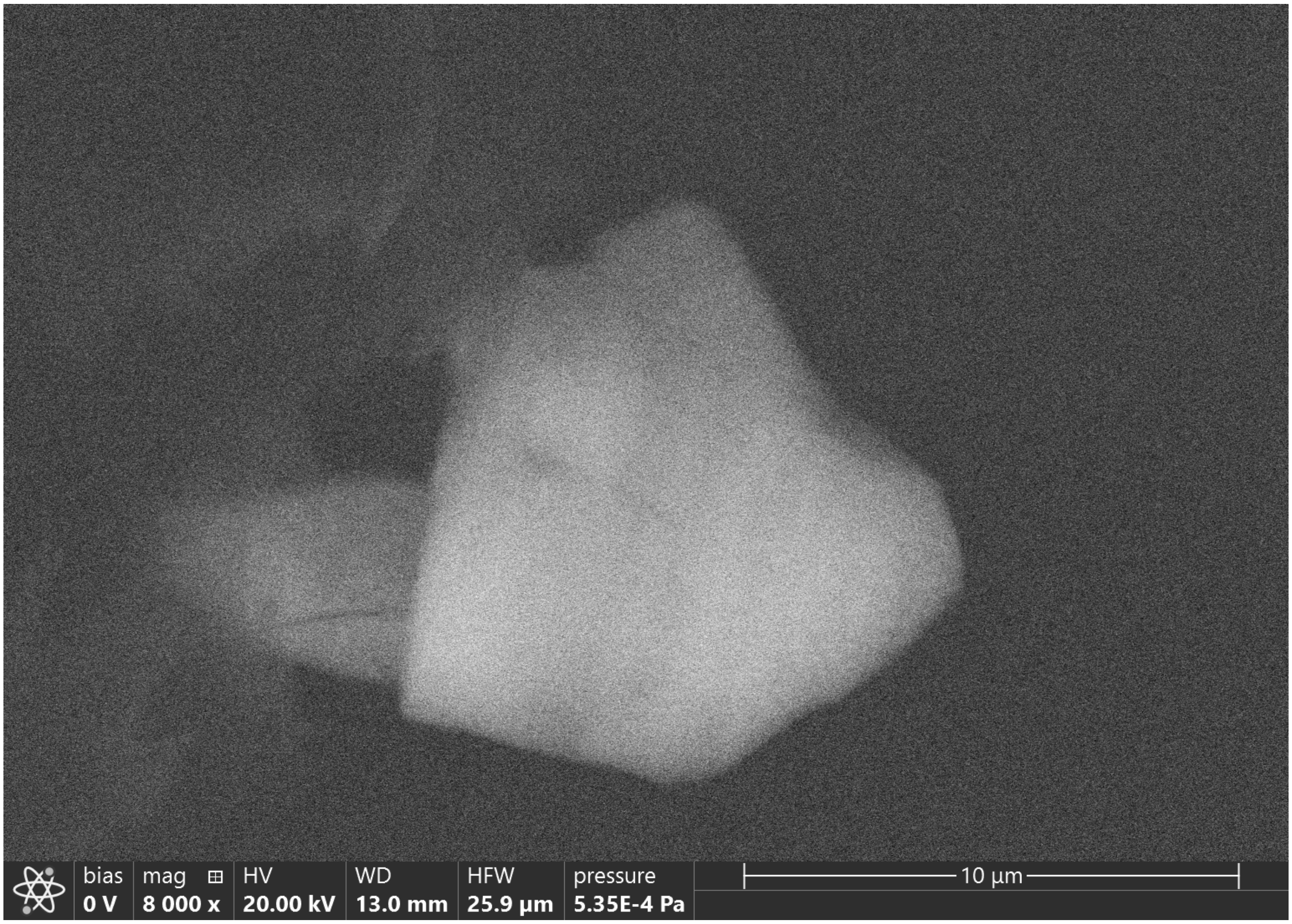


**Figure S12.** Cathodoluminescence (blue channel) of BLC preparation fabricated by the direct synthesis at 20 kV accelerating voltage.

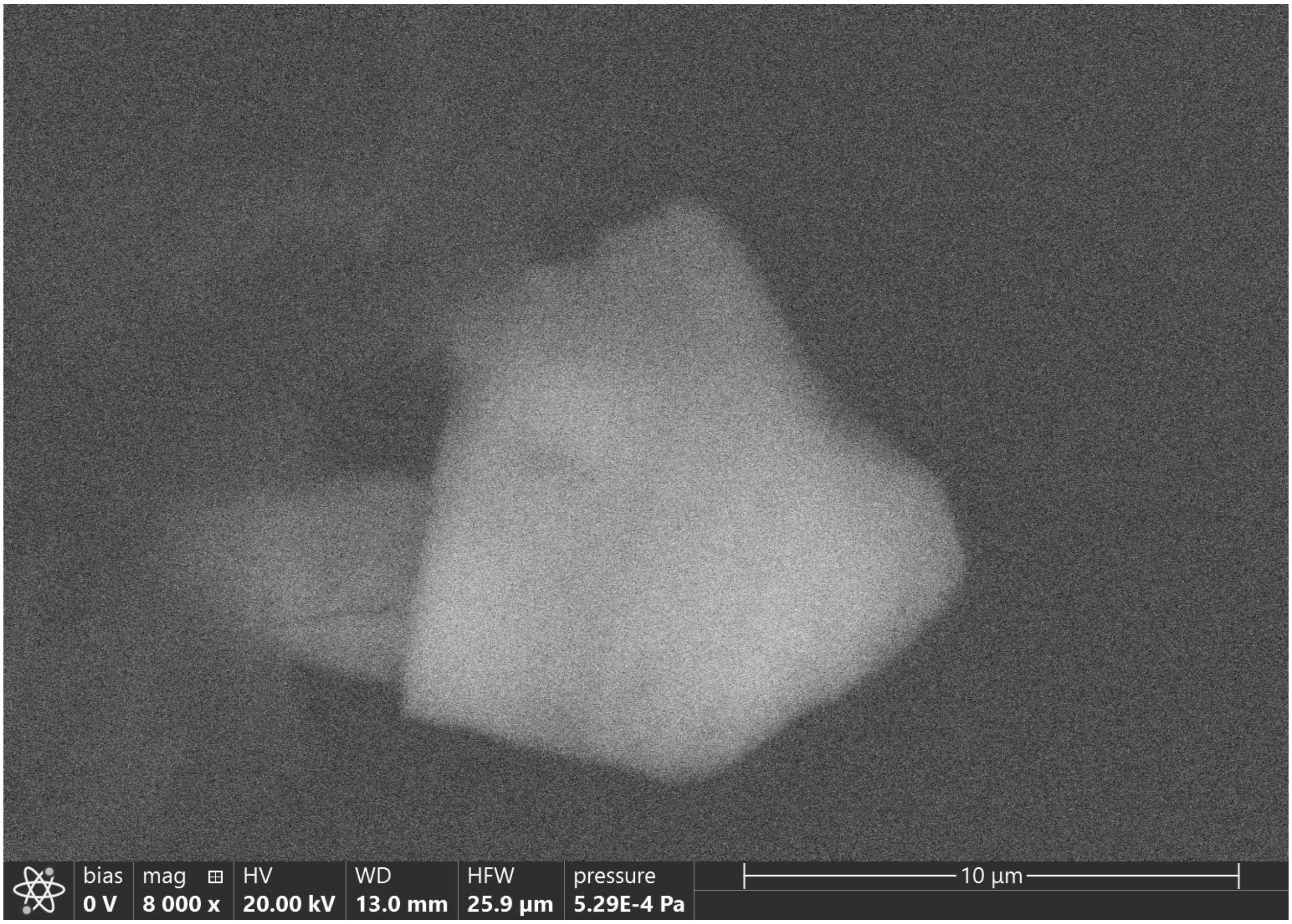


**Figure S13.** Cathodoluminescence (green channel) of BLC preparation fabricated by the direct synthesis at 20 kV accelerating voltage.

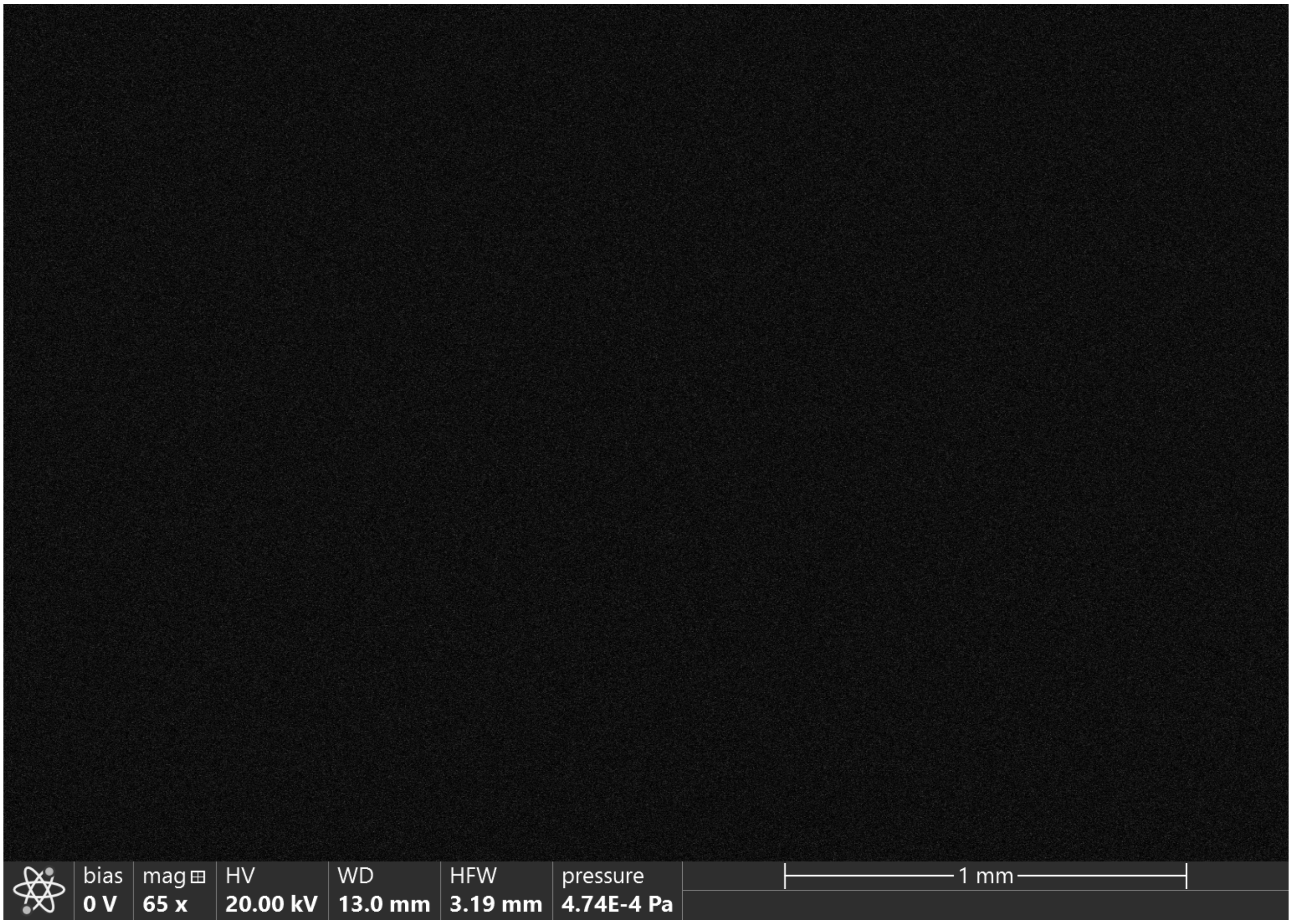


**Figure S14.** Cathodoluminescence (red channel) of BLC preparation fabricated by the direct synthesis at 20 kV accelerating voltage.
